# Preventing the onset of major depression based on the level and profile of risk of primary care attendees: protocol of a cluster randomised trial (the predictD-CCRT study)

**DOI:** 10.1186/1471-244X-13-171

**Published:** 2013-06-19

**Authors:** Juan Ángel Bellón, Sonia Conejo-Cerón, Patricia Moreno-Peral, Michael King, Irwin Nazareth, Carlos Martín-Pérez, Carmen Fernández-Alonso, María Isabel Ballesta-Rodríguez, Anna Fernández, José María Aiarzaguena, Carmen Montón-Franco, Inmaculada Ibanez-Casas, Emiliano Rodríguez-Sánchez, Antonina Rodríguez-Bayón, Antoni Serrano-Blanco, María Cruz Gómez, Pilar LaFuente, María del Mar Muñoz-García, Pilar Mínguez-Gonzalo, Luz Araujo, Diego Palao, Maite Espinosa-Cifuentes, Fernando Zubiaga, Desirée Navas-Campaña, Juan Mendive, Jose Manuel Aranda-Regules, Alberto Rodriguez-Morejón, Luis Salvador-Carulla, Juan de Dios Luna

**Affiliations:** 1 Centro de Salud El Palo, Unidad de Investigación del Distrito de Atención Primaria de Málaga Departamento de Medicina Preventiva, Universidad de Málaga, Málaga, Spain; 2 Fundación IMABIS, Unidad de Investigación del Distrito de Atención Primaria de Málaga, Málaga, Spain; 3 Mental Health Sciences, Faculty of Brain Sciences, UCL, London, UK; 4 Department of Primary Care and Population Health, UCL, London, UK; 5 Centro de Salud Marquesado, Área Nordeste de Granada, Granada, Spain; 6 Servicio de Programas Asistenciales, Gerencia Regional de Salud, Valladolid, Spain; 7 Centro de Salud Federico del Castillo, Jaén, Spain; 8 Parc Sanitari Sant Joan de Déu, Fundació Sant Joan de Déu, Barcelona, Spain; 9 Centro de Salud San Ignacio, Unidad de Investigación de Atención Primaria, Osakidetza, Bilbao, Spain; 10 Centro de Salud Casablanca. Instituto Aragonés de Ciencias de la Salud. IIS Aragón. Departamento de Medicina y Psiquiatría, Universidad de Zaragoza, Spain; 11 “Centro de Investigación Biomédica en Red de Salud Mental” CIBERSAM, Universidad de Granada, Granada, Spain; 12 Centro de Salud Miguel Armijo, Salamanca, Spain; 13 Centro de Salud San José, Linares, Jaén, Spain; 14 Unidad de Investigación de Atención Primaria, Osakidetza, Bilbao, Spain; 15 Centro de Salud Andorra, Teruel, Instituto Aragonés de Ciencias de la Salud, Teruel, Zaragoza, Spain; 16 Unidad de Investigación de Atención Primaria, Valladolid, Spain; 17 Hospital Parc Taulí, Servei de Salut Mental, Sabadell, Barcelona, Spain; 18 Unidad de Investigación de Atención Primaria, Centro de Salud Arrabal, Zaragoza, Spain; 19 Centro de Salud La Mina, Institut Català de la Salut, Barcelona, Spain; 20 Centro de Salud El Torcal, Distrito Sanitario Málaga, Málaga, Spain; 21 Departamento de Personalidad, Evaluación y Tratamiento Psicológico, Universidad de Málaga, Málaga, Spain; 22 Centre for Disability Research and Policy, Faculty of Health Sciences, University of Sydney, Sydney, Australia; 23 Departamento de Bioestadística, Universidad de Granada, Granada, Spain; 24 Departamento de Medicina Preventiva, Facultad de Medicina, Universidad de Málaga, Campus de Teatinos 29071, Málaga, Spain

**Keywords:** Depression, Primary prevention, Primary health care, Randomized controlled trial

## Abstract

**Background:**

The ‘predictD algorithm’ provides an estimate of the level and profile of risk of the onset of major depression in primary care attendees. This gives us the opportunity to develop interventions to prevent depression in a personalized way. We aim to evaluate the effectiveness, cost-effectiveness and cost-utility of a new intervention, personalized and implemented by family physicians (FPs), to prevent the onset of episodes of major depression.

**Methods/Design:**

This is a multicenter randomized controlled trial (RCT), with cluster assignment by health center and two parallel arms. Two interventions will be applied by FPs, usual care versus the new intervention predictD-CCRT. The latter has four components: a training workshop for FPs; communicating the level and profile of risk of depression; building up a tailored bio-psycho-family-social intervention by FPs to prevent depression; offering a booklet to prevent depression; and activating and empowering patients. We will recruit a systematic random sample of 3286 non-depressed adult patients (1643 in each trial arm), nested in 140 FPs and 70 health centers from 7 Spanish cities. All patients will be evaluated at baseline, 6, 12 and 18 months. The level and profile of risk of depression will be communicated to patients by the FPs in the intervention practices at baseline, 6 and 12 months. Our primary outcome will be the cumulative incidence of major depression (measured by *CIDI* each 6 months) over 18 months of follow-up. Secondary outcomes will be health-related quality of life (*SF-12* and *EuroQol*), and measurements of cost-effectiveness and cost-utility. The inferences will be made at patient level. We shall undertake an intention-to-treat effectiveness analysis and will handle missing data using multiple imputations. We will perform multi-level logistic regressions and will adjust for the probability of the onset of major depression at 12 months measured at baseline as well as for unbalanced variables if appropriate. The economic evaluation will be approached from two perspectives, societal and health system.

**Discussion:**

To our knowledge, this will be the first RCT of universal primary prevention for depression in adults and the first to test a personalized intervention implemented by FPs. We discuss possible biases as well as other limitations.

**Trial registration:**

ClinicalTrials.gov identifier: NCT01151982

## Background

### Depression as a public health problem

In the European community, major depression is the most common single mental disorder, with a 12 month prevalence of 4% [1], reaching 7% in USA [2]; and in European primary care attendees increasing to 13.9% in woman and 8.5% in men [3], or 14% in Spain [4]. A systematic review of 24 cost-of-illness studies of depression has reported that depression has substantial economic consequences for society [5]. For example, in 2004 the total annual cost of depression in Europe was estimated to be €118 billion, or €253 per inhabitant [6]. Major depression is projected to rank as the greatest contributor to illness burden by 2030 in high-income countries [7] due to its high prevalence, high impact on functioning, and early age of onset [8]. Most suicides are committed by people with depression [9], and the mortality rate of depressed patients exceeds 1.65 times that of the general population [10]. Despite effective treatments for depression, curative interventions can only reduce the disease burden of depression by 20%, because not all cases are recognized as such, and when recognized not all will receive appropriate treatment or adhere to the given treatment [11]. From a public health point of view, besides improving the whole process of depression care [12], we need comprehensive approaches to depression prevention [13].

### Risk strategies and primary prevention of depression

Primary prevention aims to reduce the incidence of new episodes of depression. When preventive measures are applied to the general population, regardless of their risk factors, primary prevention is called “*universal prevention*”. All randomized controlled trials (RCT) of universal prevention of depression have been conducted in children and/or adolescentes [14,15]. If primary prevention strategies are applied to people who have risk factors for depression, then it is called “*selective prevention*”. Here RCT have been undertaken in children and adolescents [16,17], elderly people [18,19], and in adults with specific risks for depression: stroke [20], postpartum [21-23], cancer [24], diabetes [25], macular degeneration [26], patients with complex medically illnesses [27], caregivers of relatives with dementia [28], or social risk patients [29], among others. A particular group of people at risk of depression are those with depressive symptoms, which do not meet DSM-IV diagnostic criteria for major depression. Primary prevention in this group of 105 people who are "subclinically depressed" is referred to “indicated prevention” [30-32].

### The ‘predictD’ algorithm

In recent years, our research group has developed and validated a risk algorithm (“the predictD algorithm”) to predict the onset of episodes of major depression in primary care attendees, in Europe [33] and Spain [34]. The Spanish algorithm obtained good calibration and discriminative validation [C-index = 0.82 (0.79-0.84)]. From 39 known risk factors of depression, 12 were included in the Spanish prediction model: six were patient characteristics or past events (sex, age, sex*age interaction, education, childhood physical abuse, and lifetime depression) and six were current status (SF-12 physical score, SF-12 mental score, dissatisfaction with unpaid work, number of serious problems in very close persons, dissatisfaction with living together at home, and taking medication for stress, anxiety or depression). The calculator of the likelihood of becoming depressed at 12 months is accessible at http://www.rediapp.org/predict.php. The "predictD algorithm" provides, in addition to the quantification of the overall risk of depression, knowledge of those risk factors influencing a given patient and that could guide a possible preventive intervention. This could allow us, as in cardiovascular disease, to develop interventions tailored in both intensity (level of risk) and specificity (profile of risk). This type of primary prevention might be called “*personalized prevention*” [35,36].

### Effectiveness of primary prevention in depression

There are at least 5 systematic reviews or meta-analyses [37-41] of effectiveness of primary prevention in depression in children and/or adolescents. A systematic review on Australian school-based prevention for anxiety and depression [37] included 24 RCT of 9 intervention programs (Friends, moodGYM, Aussie Optimism, etc.). Six were universal interventions, two indicated programs and one was a treatment program. Most were associated with short-term improvements or symptom reduction at follow-up. A meta-analysis (13–4 RCT) [38] of school-based cognitive-behavior interventions to prevent depression (all interventions were delivered at the group level) found that they were effective for reducing depressive symptoms at 1 and 3 months (with statistical significance), but at 6 and 12 months the differences were not significant. A meta-regression of other meta-analyses [39] (47 RCT and 32 prevention programs) found larger effects for programs targeting high-risk individuals, samples with more females, samples with older adolescents, programs with a shorter duration and with homework assignments, and programs delivered by mental health professional interventionists versus teachers. Another systematic review [40] (42 RCT, relating to 28 individual school-based programs) also found that indicated programs were most effective, and teacher program leaders less effective. The most recent Cochrane meta-analysis [41] (15–10 RCT) concluded that the risk of having a depressive disorder post-intervention was reduced at 3, 9 and 12 months; although there was significant heterogeneity. The persistence of the findings suggests that this is real and not a placebo effect.

A recent meta-analysis (20 RCT) [42] focusing on prevention of postpartum depression, reported a Relative Risk of 0.68 (95% CI: 0.66-0.93). Promising interventions included the provision of intensive, professionally-based postpartum home visits, telephone-based peer support, and interpersonal psychotherapy. A meta-analysis [43] of psychosocial preventive interventions to reduce depressive symptoms in low socio-economic-status women (14 RCT) found an overall effect size of 0.31(95% CI:0.17-0.45). A meta-analysis (4 RCT) [20] identified a significant effect (Odds Ratio:0.64; 95% CI:0.42-0.98) of psychotherapy for preventing depression after stroke, but there is no evidence of efficacy of antidepressants in preventing depression (10 pharmaceuticals RCT) [20].

On the other hand, there are 4 meta-analyses [44-47] that mixed different types of populations. The first [44] evaluated 7 RCT of psychological treatments for patients with subthreshold depression. The relative risk of developing a major depressive disorder in subjects who received the intervention was 0.70 (95% CI: 0.47-1.03). The second [45], combining 6 RCT that used cognitive-behavioral training called “coping with depression” to prevent depression, found an Incidence Rate Ratio (IRR) of 0.62 (95% CI: 0.43-0.91). The third [46] (19 RCT and 21 comparisons) included different types of prevention (universal:2, selective:11 and indicated:8), age (adolescents: 9 and adults:12), interventions (cognitive-behavioral:15, interpersonal:3 and others:3), format (groups:18 and individual:3), target group (postpartum:7, school:6 and other:8), and type of prevention (universal:2, selective:11 and indicated:8). The combined IRR was 0.78 (95% CI: 0.65-0.93). A fourth meta-analysis [47] adding 11 further RCT to the previous meta-analysis [46] reported an IRR of 0.74 (95% CI: 0.65-0.85).

### Justification

Data suggest that interventions to prevent depression are effective, although this effect seems small or moderate and there are a number of limitations: 1) a lack of evidence on longer term follow-up; 2) many RCT had samples of insufficient size to find significant differences for the incidence of new depression cases; 3) data suggest that universal prevention has a lower effect than selective and indicated prevention, although this affirmation is only applicable for school-based interventions, since no RCT has applied universal prevention in adults; and 4) there are no conclusive data on the superiority of any one intervention. On the other hand, cost-effectiveness studies on prevention of depression are scarce [48-50], so further investigations are needed to decide on the general implementation of primary prevention programs for depression [51,52].

Most primary prevention of cardiovascular diseases is performed in primary care and the community, while specialists (cardiologists, endocrinologists, nutritionists, etc.) play a role more focused on complicated and serious cases. This is also the case in depression. The target for primary prevention of depression is the healthy person and thus it is reasonable that primary prevention of depression is carried out in primary care and the community. However, few RCT of primary prevention of depression are conducted in primary care [28,29,32], and all the interventions were implemented by specialists in mental health (therapists, psychologists, psychiatric nurses, etc.).

We aim to conduct a cluster randomized trial in primary care with a new intervention to prevent the onset of major depression, based on the level and profile of risk of depression (personalized prevention), involving adult patients at low, moderate and high risk as measured by a risk algorithm (universal prevention) and implemented by family physicians (FPs). We will recruit a sample of non- depressed primary care patients that is large enough to detect a significant reduction in the incidence of new episodes of depression over 18 months. Our main outcome will be the effectiveness of this new intervention, but we will also assess its cost-effectiveness and cost-utility.

## Objectives

The main objective is to measure the effectiveness of a new intervention for primary prevention of major depression based on the level and profile of risk of primary care attendees. Among the secondary objectives are to evaluate the cost-effectiveness and cost-utility of the intervention versus usual care.

## Methods/design

### Design

A multicenter randomized controlled trial, with cluster assignment by health center and two parallel arms. Two interventions will be applied: usual care in the control group and a new intervention for primary prevention of major depression based on the level and risk profile of patients. These interventions will be applied by the FPs. The main outcome is the cumulative incidence of major depression during the follow-up, with evaluations at baseline, 6, 12 and 18 months. Inferences will be made at the patient level.

The predictD-CCRT study is in compliance with the Helsinki Declaration. The predictD-CCRT study has been approved by the relevant ethics committees in each participating Spanish city: Ethics Committee on Human Research of the University of Granada, Ethics and Research Committee of Primary Health District of Malaga, Ethics Committee for Clinical Research of Sant Joan de Deu Foundation (Barcelona) (PIC CEIC-62-09), Ethics Committee for Clinical Research of Aragon (CEICA) (CP06/05/2009), Ethics Committee for Health Research of the Jaen Hospital, Ethics Committee for Clinical Research of Euskadi (CEIC-E) (03/2009), Ethics Committee for Clinical Research of the Rio Hortega Hospital of Valladolid (04/2009).

### Setting

Health Centers in seven Spanish cities will participate: Malaga, Jaen and Granada in southern Spain; Valladolid in western Spain; Saragossa and Bilbao in northern Spain; and Barcelona in eastern Spain. Each health center covers a population of 15,000 to 30,000 inhabitants from a geographically defined area. The FPs in each health center work as a group, with extensive primary care teams. The Spanish National Health Service provides free medical cover to over 95% of the population. Patients can visit their doctors as often as they want without having to pay for it, even when they do so for preventive reasons. Each patient is assigned to only one FP, who has gatekeeper functions.

### Sample selection of participants and exclusion criteria

#### Health centers

From a possible total of 220 health centers in the 7 participating cities, those that do not have electronic clinical charts or have had them for less than 2 years or plan to change them in the next 18 months will be excluded. All others will be invited to participate. From those centers that agree to participate, a random sample of 10 health centers per city, 70 health centers in total, will be selected. These will then be randomized by an independent person from the research group at the coordinating center of the study (Malaga), using closed and opaque envelopes.

#### Family physicians

In each participating health center we will exclude those FPs who are unfamiliar with using the clinical chart or are planning to change their place of work in the next 18 months. We shall then invite all remaining FPs to participate and from those who agree, randomly select two per center (before randomization of centers) using closed and opaque envelopes selected by an independent person from the research group. This will mean 140 FPs will participate in the trial.

#### Patients

Patients belonging to the 140 FPs will be selected using a systematic random sampling, each 4–6 patients, from the FPs’ appointment lists at random starting points for each day. This will be done by an assistant researcher for each health center. The list of selected patients will be given every day to the family physician (FP) before starting to see patients. FPs will check the selected patients to see if they meet any of the following exclusion criteria: age under 18 or over 75 years; inability to understand or speak Spanish; severe mental disorder (psychosis, bipolar, personality disorder,…); cognitive impairment; terminal illness; the patient is scheduled to be out of the city more than four months during the 18 months of the follow-up; and persons (representatives) who attend the surgery on behalf of the person who has the appointment. The FPs will introduce the study to the selected not excluded patients and will request permission before contacting the assistant researcher. Patients who refuse to participate will not be replaced, prolonging the days of recruitment to achieve 26–27 patients for each FP. Those who give informed consent will undertake a research interview within two weeks with the Composite International Diagnostic Interview (CIDI) in order to detect the presence of major depression. Patients with a diagnosis of current major depression will also be excluded from the trial. The exclusion criteria for health centers, FPs and patients are shown in Table 1.

**Table 1:** Exclusion criteria of the predictD-CCRT study

***Health centers***	
•	Do not have electronic clinical charts or have had them for less than 2 years
•	Plan to change their electronic clinical charts in the next 18 months
•	No consent to participate in the study
***Family Physicians***	
•	Unfamiliar with using the clinical chart
•	Planning to change their place of work in the next 18 months
•	No consent to participate in the study
***Patients***	
•	Age under 18 or over 75 years
•	Inability to understand or speak Spanish
•	Severe mental disorder (psychosis, bipolar, personality disorder…)
•	Cognitive impairment
•	Terminal illness
•	The patient is scheduled to be out of the city more than four months during the 18 months of follow-up
•	Persons (representatives) who attend the surgery on behalf of the person who has the appointment
•	Diagnosed with major depression by the CIDI*
•	No consent to participate in the study

* Composite International Diagnostic Interview.

### Randomized allocation

The randomization and allocation to the arms will be carried out according to the health center; that is, all FPs and patients from one health center will be included in the same arm. If the same health center has FPs in both the control and the intervention groups, the likelihood of the FPs in the control group becoming contaminated by the intervention group is high, since Spanish health centers generally work as a team. We aim to evaluate a new intervention for primary prevention of major depression, which in principle will be delivered at patient level by the FP; however, due to the nature of the intervention some components could be delivered at community level, so the contamination between patients in the control and intervention groups in the same health center is likely. Therefore we have decided on cluster randomization to avoid this possibility in patients and FPs.

The randomization of the 70 health centers to allocate them to the control or intervention groups will be done stratifying by city. Thus, we will have 35 health centers assigned to the control group and 35 to the intervention group, 5 and 5 respectively for each one of the seven cities participating in our study. The randomization will be undertaken centrally, from the coordinating center in Malaga, by an independent person outside the research group using closed and opaque envelopes.

### Masking

In trials that evaluate psychosocial interventions it is not possible for professionals who provide the new intervention or for patients who receive it to be blind to it [53]. However, in our trial those who assess outcomes (interviewers) will be different and independent from those who provide the intervention (FPs). Moreover, the interviewers will be not informed of the patient’s status in the control or intervention group. Those who perform statistical analyses will also be blind to the intervention and control codes.

### Sample size

The sample size was calculated assuming that the cumulative incidence of depression in the control group will be 12% [34] while the incidence in the intervention group will be 5 points below that 12%. The Type I error of the chosen contrast was 5% and the power 80%. The sample size obtained assuming a simple random sample would be 430 people in each group. As we will undertake a cluster randomization (by health center), and decided to have 35 health centers in the control group and 35 in the intervention group (48 patients in each health center), we obtained an effect design of 3.82, with the assumption that the intraclass correlation coefficient of the health center is 0.06 [34]. Thus we need a sample size of 1643 patients in each trial arm, making a global sample of 3286 patients, 140 family doctors and 70 health centers. These calculations assume that the distribution of 2 FPs per health center and 24 patients per FP will be very homogeneous (coefficients of variation of cluster sizes <0.15). We also expect to increase the sample of patients recruited by FPs from 24 to 26–27, since about 10% of potential patients will have a diagnosis of major depression on CIDI at baseline and will need to be excluded.

### Follow-up

All patients enrolled in the trial will be evaluated at baseline, 6, 12 and 18 months. Interviewers trained and independent from the FPs providing usual care or the new intervention will administer the CIDI and the other questionnaires. FPs participating in the trial will complete a self-administered questionnaire at baseline.

### Variables

#### Main outcome

Our primary outcome will be the cumulative incidence of major depression during 18 months of follow-up. We will use the section of depression of the CIDI [54,55], developed and validated by the WHO [56]. The CIDI is a fully structured diagnostic interview that provides current diagnoses according to DSM-IV categories. We will use it to estimate the onset of major depression in each prior 6-month period (0–6, 6–12, and 12–18 months). Interviewing in this way, at baseline, 6, 12 and 18 months, we will obtain the most accurate picture of the cumulative incidence of major depression.

#### Secondary outcomes

As secondary outcomes we will use measurements of health-related quality of life using the 12-item Short Form (SF-12) [57,58] and the EuroQol [59-61] with evaluations at baseline, 6, 12 and 18 months. Furthermore, we will make estimates of cost-effectiveness and cost-utility (see Economic evaluation below).

#### Independent variables

##### Patient variables

• Socio-demographic characteristics: province, sex, age, marital status (married/living with partner, separated, widowed, divorced or single), employment status (employed, unemployed/looking for a job, retired, unable to work, looking after family or home, in full-time education, and other), educational level (beyond secondary education, secondary education, primary education and incomplete primary education/illiterate), owner-occupier of an accommodation (owner, mortgage, rented, and other), living alone or with others [62].

• Anxiety disorders using the anxiety section of the Primary Care Evaluation of Mental Disorders (PRIME-MD) [63]. The Spanish version of the PRIME-MD can classify patients who test positive for panic attack, generalized anxiety disorder and other anxiety disorders [64]. We will use a dichotomous anxiety variable to indicate when any of the three diagnoses of anxiety are present in a given patient.

• Controls, demands and rewards for unpaid work, using an adapted 7 item version of the job content instrument [65]. From the sum of the seven items, the variable is categorized in 3 (satisfied, dissatisfied and very dissatisfied).This questionnaire has previously shown good validity and reliability in Spain [62].

• Satisfied with living together at home (5-Likert response options).

• A lifetime screen for depression based on the first two questions of the CIDI [66].

• Childhood experiences of physical abuse (5-Likert response options) [67].

• Presence of serious physical, psychological or substance misuse problems, or any serious disability, in persons who are close friends or relations of participants. These questions can be used as 4 different items (yes/no) or as an ordinal variable (summation of the 4 questions).

• Whether the participant’s mother committed suicide (yes/no) [68].

• Perception of safety inside and outside the home using a question from the Health Surveys for England with 5-Likert response options [69].

• Experiences of discrimination on the grounds of sex, age, ethnicity, appearance, disability, sexual orientation, and others using 7 questions (yes/no) from a European study [70]. The answers to these seven items can also be joined in an ordinal variable.

• Taking medication for anxiety, depression or stress in the previous 6 months (yes/no).

• Probability of the onset of major depression at 12 months, obtained from the equation “predictD-Spain”, which has been previously validated [34] and is derived from a combination of some of the above variables.

We will assess all these variables and questionnaires at baseline, 6, 12 and 18 months. The test–retest reliability of questions used in the predictD studies has been reported previously [62,71].

##### Family physician variables

The FP variables will be collected through a self-administered questionnaire at baseline:

• Age and gender.

• **Job characteristics**: urban or rural clinic, type of contract (fixed or temporary/substitute), months working at the current Health Center, list size and mean time per visit.

• **Training**: year medical degree, time working as FP (in months), postgraduate training (3 or 4 years) in family medicine (yes/no), accreditation as a FP tutor (yes/no), experience in training FPs [resident 1^st^ year assigned (yes/no), and/or resident 3^rd^ or 4^th^ year assigned (yes/no)], and membership of Spanish Health & Communication group (yes/no).

• **Satisfaction with management of patients with mental health disorders in primary care**: degree of comfort in the use of antidepressants (5-Likert response options), satisfaction with communication and care shared with the mental health team (secondary care) (5-Likert response options), satisfaction with the role of primary care nurse in mental health disorders (5-Likert response options) and satisfaction with the role of primary care social worker in mental health disorders (5-Likert response options or “I do not have social worker in my health center”).

• **Profile of professional practice**: A three-dimensional questionnaire about professional satisfaction (4 items), workload perception (4 items), and biomedical vs. psychosocial orientation (4 items) validated in Spain [72].

• **Personality** according to the Spanish version of the Eysenck Personality Questionnaire-Revised (EPQR-A) [73-75], which explores three dimensions: extraversion, neuroticism, and psychoticism.

##### Health center variables

From administrative records we will collect at baseline the following variables: inhabitants of the health center area (Basic Zone Area), size of the population where the health center is located, number of professionals (also by type of professionals) in the health center, population/professional ratios, and time of functioning as health center.

### Statistical analysis

In line with recommendations by Groenwold [76], we will perform effectiveness analyses in the following ways: analysis with covariate adjustment and 2) intention-to-treat analysis by multiple imputations with covariate adjustment. We will conduct all analyses using Stata, release 12.1 [77].

#### Multiple imputations

We will use multiple imputations by chained equations [78] by means of the ‘ICE’ program [79]. We will choose a number of imputations to limit the loss in power (no more than 1%) for testing the association of interest [80]. We will undertake a sensitivity analysis to check the changes in the estimates and variances with progressive increases in the number of imputations [78]. We will explore the plausibility of the missing at random assumption and will include in the imputation models any covariate of interest that is predictive of missingness [81]. When we have to impute a covariate, we will also include the outcome variable in the imputation model. Standard errors will be calculated using Rubin’s rules [82] which take account of the variability in results between the imputed datasets, reflecting the uncertainty associated with the missing values.

#### Regression models

We will perform multi-level logistic regressions with the cumulative incidence of depression at 18 months as the dependent variable. To test the hierarchical data structure we will use the likelihood-ratio test of the null model with health center as a random factor versus usual logistic regression. Then we will check the likelihood-ratio test of the null model with health center and FP as random factors versus the null model with only health center. We will also calculate the intraclass correlation coefficients for health center and FP.

We will include in the models the group variable (control and intervention) and will adjust for the probability of the onset of major depression at 12 months measured at baseline [34]. Where appropriate we will adjust for unbalanced variables at baseline not included in the equation to predict the onset of major depression [34]. We also will retain in the model the variable city because of an a priori assumption of clustering effect within city, although it has few categories (n = 7) that could be considered as random factors [83]. From the final model we will obtain the adjusted odds ratio (OR) of the group variable and the adjusted number needed to treat, $\mathrm{NNT}=\frac{1}{\mathrm{lo}\left(1-\mathrm{OR}\right)}$ [Io = cumulative incidence in control group], to prevent a case of major depression.

#### Inverse probability weighting

For each patient the probability of remaining in the follow-up at 18 months will be obtained through a logistic regression model (multilevel if appropriate) with the variable ‘remaining in the follow-up’ (yes/no) as dependent variable and a set of predictor variables measured at baseline. For the effectiveness analyses with complete cases we will apply the inverse probability weighting to the final model to adjust for a possible attrition bias due to participants lost during the follow-up [84,85], implemented through the ‘GLLAMM’ program [86].

### Economic evaluation

The Economic Evaluation will be presented from two perspectives: 1) a Societal Perspective, including the costs of all types of health services (direct costs) and the costs that stem from production losses (indirect costs); and 2) Health System (including only direct costs). Although NICE [87] recommends just performing economic evaluations from the Health System perspective, we will present both due to the major impact that depression has on productivity [6,88,89]. The time frame of this study will be 18 months. Therefore, we will discount both costs and effects at 3.5% following NICE recommendations [87]. All costs will be expressed in euros (€) for the reference year 2012.

#### Cost

We will use a modified version of the Client Service Receipt Inventory (CSRI) [90] to collect information about use of health care resources, use of psychotropic drugs (antidepressants, anxiolytics and hypnotic-sedatives) and lost productivity.

#### Unit costs

Direct health costs will be calculated by multiplying the number of health service units (consultations, hospital days, etc.) by their standard cost price. This unit cost will be retrieved from ‘Oblikue dataset’ (http://www.oblikue.com/). Cost of medication will be calculated by multiplying cost price per daily dose, multiplied by the number of prescription days recommended, as recalled by the patient. Information about medication costs will be obtained from the Spanish Pharmaceutical Vademecum (http://www.vademecum.es/). Indirect costs consist of the costs of absenteeism from paid work. Costs of work loss will be calculated by multiplying the days on sick leave by the minimum daily wage in Spain according to the human capital approach.

Intervention costs will include only the printed leaflet. However, we will also identify primary care visits related directly to the predictD-CCRT intervention.

#### Health effects

The difference in the cumulative incidence of depression at 18 months will be measured as stated in the paragraph described above (‘main outcome’). Quality Adjusted Life Years. (QALYs) will be measured using the EuroQol-5D. Spanish tariffs will be used to estimate the utility of health states described by the patients [91,92]. QALYs will be calculated by multiplying the utility by the amount of time a patient spent in a particular health state. Linear interpolation will be used for transitions between health states. QALYs will be assessed at baseline, 6, 12 and 18 months.

#### Cost effectiveness and cost-utility analysis

As in the effectiveness analyses, we will take two approaches to the cost-effectiveness and cost-utility analyses: 1) a complete case analysis with covariate adjustment and 2) an analysis after multiple imputations with covariate adjustment. For the first strategy we will apply the inverse probability weighting to the final model. We will calculate incremental cost-effectiveness ratios (ICER), which are calculated as incremental cost (mean cost for intervention group minus mean cost for control group) divided by incremental effectiveness (mean effect for intervention group minus mean effect for control group) [93]. Incremental cost-utility ratios (ICUR) will be calculated in the same way. The only difference is that the effect will be the difference in QALYs. The incremental costs and incremental health effects will be modeled by generalized linear models (GLM). The modified Parks test will be used in order to select the appropriate family. In order to identify the correct link function we will compare model performance of all permutations of candidate link and variance function using different diagnostic tests such as the Pregibon Link test, the Hosmer-Lemoshow test and the Copas test [93]. We will check the intraclass correlation coefficients and the likelihood-ratio tests with health center, FP and both as we said before. If the clustering effect is relevant, we will use robust standard errors for the cluster indicated.

#### Quantification of uncertainty

To estimate the uncertainty around the ICER and ICUR, we will calculate 95% confidence intervals around the mean cost differences using the non-parametric confidence interval. Bootstrapping with 5000 replications will be performed on each imputed dataset [93,94].

#### Generation of cost-effectiveness planes and cost-effectiveness acceptability curves

Bootstrapped cost effect pairs will then be plotted on cost effectiveness planes and used to estimate cost effectiveness acceptability curves (CEACs). In the cost-effectiveness planes the ‘x’ axis represents the difference in effects and the ‘y’ axis the difference in costs. CEACs show the probability that a treatment is cost effective at a specific ceiling ratio, which is the amount of money society is willing to pay to gain one extra unit of effect. Willingness to pay values will range from 0€ to 100,000€ [95].

#### Sensitivity analyses

A number of sensitivity analyses will be conducted in order to assess the robustness of the results. Among others, these analyses will include: modification of the unit cost of different values such as primary care physician visits or nurse practitioner; modification of discount rates (from 0% to 6% as NICE recommends); modification of the cost for absenteeism (using the mean salary instead of the minimum); and modification of the cost including loss of productivity by presenteeism.

## Intervention

The new intervention will be applied at patient level by the FPs in the intervention group. This intervention will be tailored to each patient based on his/her risk profile of depression (risk factors present) and his/her risk level (likelihood of becoming depressed at 12 months), and it will be built up from a total of 5 components, which may act both independently and interdependently [96]:

**Figure 1:**
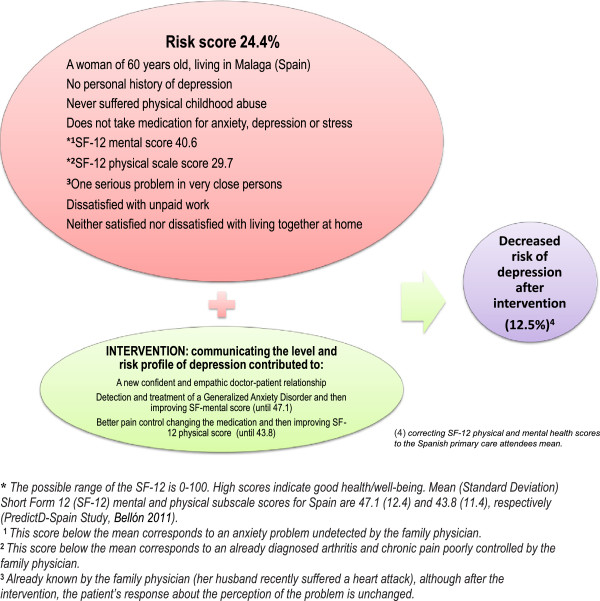
Example of the mechanisms to reduce the likelihood of becoming depressed after the intervention to prevent the onset of depression by the family physician.

1) *Training workshop for family physicians in the intervention group*

A qualitative research study conducted prior to this RCT [97] found that FPs were resistant to informing their patients about their risk of depression, and also raised doubts about the validity of the predictD risk algorithm. FPs said they had little training on prevention of depression, and expressed uncertainties about what they should advise their patients. They were also fearful about the possible distress that could result in patients being informed of their risk for depression and commented on the lack of time for these preventive activities. Accordingly, the FPs will receive a 10–15 hour training workshop during working hours in the new intervention. This will enable them: 1) to interpret and make a specific and global evaluation of the risk for depression for each patient; 2) to transmit this information to the patient in an understandable and comprehensible way, without causing alarm; 3) to undertake active listening to patients about their beliefs, expectations and impact on information transmitted; 4) to provide tailored advice for each patient depending on his/her set of modifiable risk factors for depression; 5) to be able to integrate and personalize advice to give each patient based on his/her health history, previous doctor-patient relationship and bio-psycho-family-social circumstances; 6) to support those patients excessively worried by the information given. In the workshop we will use role-playing and video comments in groups in order to discuss and practice with FPs how to deal with different situations. The educational units, that we will use, are shown in Additional file 1: Annex 1. Previously, we will “train the trainers” to ensure that the training of the FPs will be performed to a minimum standard of quality and in standardized manner in each of the cities participating in the study.

2) *Communicating the level and risk profile of depression*

How patients are informed about their risk of becoming ill (cardiovascular events, hip fractures…) determines the outcome of prevention efforts [98,99]. In the same qualitative study as mentioned above, we found that primary care patients are pleased to be informed of their risk for depression [97]. Communicating with patients about their level and risk profile for depression is very new as until now there have been no validated risk algorithms to predict its onset [33,34]. This is also “*the starting component*”, which will activate the other components of the intervention. Each FP will receive (at baseline, 6 and 12 months) a report with the information on risk from each of 24 patients not depressed at baseline (belonging to their usual patient list) who will be randomly selected and will provide informed consent to participate in the study. This report will include the likelihood of becoming depressed at 12 months and the patients’ responses to each of the risk factors included in the predictD-Spain equation [34]. An example of a report of a patient who has 24.4% of becoming depressed at 12 months is shown in Figure 1.

If a patient has a moderate or high risk level (2nd or 3rd tertile), the FP will arrange an appointment with the patient aiming to communicate his/her risk of depression. If a patient has a low risk (1st tertile), the FP will have the option to report by telephone or face to face depending on the patient’s preferences. If a visit is scheduled, this will last approximately 10 minutes. This process of transmitting risk information will be held at baseline, 6 and 12 months. Reports will not include information about major depression diagnosis by CIDI at 6 and 12 months; although we will recommend that FPs screen for depression if the risk of depression was moderate or high at 6 and 12 months. FPs will write down on the problem list (clinical chart) the label “*predictD*” in order to remind them on opening their clinical charts that patients are included in the program predictD to prevent depression. This will help to ensure that when patients see their FPs, regardless of the reason for the consultation, doctors will take into account the information on their risk of depression if applicable.

3) *Building up a tailored bio-psycho-family-social intervention to prevent depression by family physicians*

Unlike psychosocial interventions developed to date to prevent depression, our intervention will be provided by FPs. With this approach we intend to draw on and integrate those usual components of primary care that could converge in the prevention of depression:

1. Previous knowledge and doctor-patient relationship: Family physician’s commitment to the patient has no defined end point. This continuity of the relationship and attention over time helps FPs to acquire a comprehensive understanding of the health problems, illness behavior, coping style, cultural background, and family and social context of their patients. Moreover, most patients will develop trusting relationship with their FPs. Patients will feel free to expose personal aspects of their lives in order to receive help, and FPs will need less time to know and understand particular aspects of any situation.

1. Establishment of a basic psychotherapeutic relationship: Many patients already consider FPs to be their primary source of mental care [100], although many FPs are not aware of this. Patients expressed this idea when asked about who would be the most appropriate person to communicate their risk of depression [97]. An interview in which a FP tries to communicate adequately to the patient his/her level and risk profile of depression will encourage the patient to express his/her concerns about the circumstances and problems that, in his/her opinion, could precipitate depression. FPs will then actively listen and offer an emphatic response, gathering additional and relevant information where needed and giving any advice if appropriate. The patient and the FP could agree to schedule a new visit to continue talking about preventing depression, but the only mandatory visits that FPs will arrange are those to report the risk of depression at baseline, 6 and 12 months. These are not psychiatric interviews or formal psychotherapeutic interventions, but may give the opportunity for the patient to feel heard and understood from a cognitive and emotional standpoint [101]. It is also intended to help the patient to be activated and empowered.

1. Family-oriented practice: FPs often bring a family orientation to bear in their clinical practice [102]. It has been reported that there are up to 5 levels of family-centered medical care in primary care [103]. We believe that the FP in our study are at the third level: FPs take an active interest in the feelings and concerns of the family in an empathic way. Family-oriented practice should be understood from the system theory, so that a change in any element of the family system could prevent depression in the patient. For example, a FP could help to improve the quality of life of a person who has the responsibility of caring for a relative with Alzheimer’s Disease and this may prevent depression [28]. This is feasible because in Spain members of the same family usually share the same FP. Primary care patients think it is useful to involve the family and consider the family resources and the possibility of involving other family members in coping with the risk of depression [97].

1. Social prescribing and community referral by FPs: Many social and personal problems cannot be dealt with effectively by the FP and require community resources [104]. The social dimension of the practice of primary care is the least accepted by FPs [105] and little is known about the effect on health of social prescribing and community referral by the FP. However, we will encourage FPs in the intervention group to become familiar with the community resources in their area. We will advise them on using centers. Moreover, we will advise them on using social prescribing in patients deemed appropriate with the aim of preventing depression. Primary care patients suggested participation in informal support groups to help them restructure their lives [97].

1. Management of physical problems: some physical problems are considered risk factors for depression, such as serious diseases (cancer, heart attack, stroke, …) [20,24], disabling illness (blindness, deafness, arthritis, …) [26] and/or chronic health problems (diabetes, chronic pain, …) [25]. Moreover, in our predictD studies [33,34] poor physical quality of life was an important predictor of depression, and FPs will receive this information in the patient’s reports. Therefore, appropriate management of physical problems could also help prevent depression.

4) *Offering a booklet to prevent depression*

At the first intervention visit, at baseline, FPs will give patients a brief booklet (a diptych) with advice for preventing depression (Additional file 1: Annex 2).This booklet is based on the recommendations on health promotion and preventive activities of the " Spanish Family and Community Medicine Society", in its section on mental health (PAPPS) [106]. At the end of this booklet reference is made to three websites with information for patients and relatives about depression, anxiety and insomnia [107].

5) *Activating and empowering patients*

People have favorable attitudes and beliefs about prevention of depression that do not conflict with evidence-based programs [100,108]. Activating and empowering patients to act on this knowledge might lead to an increased perception of self-efficacy, which is the gateway to changing attitudes and behavior to prevent depression. Primary care patients are in favor of receiving information about their risk for depression from their FP but seeking solutions themselves, with or without the help of the FP [97]. We will encourage FPs to invite patients to make suggestions during the interview on strategies, attitudes and behaviors they are already using to prevent depression. FPs will then positively reinforce those that in their opinion are the most appropriate to prevent depression for each patient.

### Explanatory model for the prevention of depression in primary care

**Figure 2:**
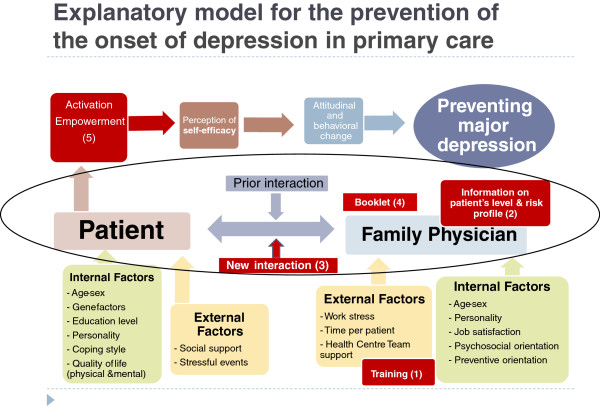
**Explanatory model for the prevention of the onset of depression by family physicians.** Component 1 (training of family physician) prepares the family physicians for the intervention and component 2 (communicating the level and risk profile of depression to the patient) initiates it. This leads to a new doctor-patient interaction, which triggers component 3 (building up a tailored bio-psycho-family-social intervention to prevent depression by family physicians) that activates and empowers the patient (component 5), and which increases the patient’s perception of self-efficacy. This can also be achieved by component 4 (offering the patient the booklet) at the first doctor-patient interview.

Figure 2 shows the theoretical model that integrates the five components of the intervention to prevent depression from primary care: There are patient’s internal risk factors (sex, age, gene, personality, etc.) and external factors (threatening events) that can trigger an episode of major depression, depending on internal (coping style) and external (family and social support) resources that might be activated. Firstly, we introduce in the system “the training workshop” for FPs (*component 1*) to improve their knowledge, attitudes and skills to prevent depression with the new intervention. The FP usually has previous knowledge and a doctor-patient relationship (*component 3.1*) that generally predisposes toward a better intervention to prevent depression by both doctor and patient. We will give FPs risk information from patients, which they will use to communicate their level and profile of risk of depression (*component 2*). This is the “starting component” that will activate the rest of the components, creating a new doctor-patient interaction, whose essential element is the establishment of a basic psychotherapeutic relationship (*component 3.2*). The family-oriented practice (*component 3.3*), social prescribing and community referral (*component 3.4*), and management of physical problems (*component 3.5*) might be activated or not depending on the risk factors of depression involved in each patient, the FP’s skills, and the patient’s preferences. In all cases, FPs should provide the booklet to prevent depression (*component 4*), although the patient may or may not decide to use it. Finally, the FPs, taking into account the above components, will encourage their patients to speak about those strategies, attitudes and behavior they are already doing to prevent depression, in order to achieve their activation and empowerment (*component 5*). Thus, the patients can improve their perception of self-efficacy, which in turn promotes changes in attitudes and behaviors to prevent depression. There are also internal (sex, personality, psychosocial and preventive orientation, etc.) and external (work stress, team support, etc.) factors in the doctors that may influence their ability to conduct a successful intervention. The differences between interventions to prevent depression conducted so far in adults and the new intervention that we propose in primary care are summarized in Table 2. An example of how the intervention can lead to a reduced risk for depression is shown in Figure 1, and another example on the doctor-patient interview to prevent depression can be seen in a video (Additional file 1: Annex 3).

**Table 2:** Differences between interventions to prevent the onset of depression evaluated so far in adults and the new intervention in primary care

**Characteristics**	**Other interventions**	**New intervention**
**Type of prevention**	Selective or indicated^1^	**Universal**^2^
**Orientation**	Psychosocial (cognitive-behavioral, interpersonal…) or psychoeducational	**Biopsychosocial** (primary care orientation) and based on level and risk profile of becoming depressed (**personalized**)
**Type of intervention**	Formal (fixed rules)	Not formal (**tailored rules**)
	Same number of sessions for all patients	Minimum of three interviews but no maximum
	Same components for all patients	Number of components involved tailored
**Who implements the intervention?**	Therapists or specialists in mental health (psychologist, psychiatric nurse…)	**Family physicians**
**Sample size**	Small	**Large**

^1^ Patients with a specific risk of depression, high risk or subclinically depressed.

^2^ Patients with a low, moderate or high risk of depression. To date universal interventions have been evaluated to prevent depression but only in children and adolescents.

### Quality control of the intervention

In order to control the satisfaction and adherence to the intervention we will undertake two quality controls. The first one will be from a subjective focus. After each interview to inform patients about their risk of depression, the FPs will have to answer four questions: Have I written the label “predictD” in the clinical chart? (yes/no); what is my level of satisfaction with this interview to report the risk of depression? (5-Likert response options); would I change anything in this interview? (open answer); and observations (open answer). All patients will be asked about their overall satisfaction with their doctor (5-Likert response options); and only patients receiving the intervention will respond to the question “What is your level of satisfaction with the last interview in which your doctor informed you about your risk of depression?” (5-Likert-5 response options or “I was not informed”). The second is from an objective focus: we will review the clinical charts to confirm that FPs have included the label “predictD” on the problem list and we will also collect the number and content of visits related to the intervention.

### Side effects of the intervention

It is possible that some patients will react with fear or worry when receiving information about their level and risk profile of depression, especially apprehensive patients or those that already have a high level of anxiety. If a patient does not want to receive this information he/she will not sign the consent and therefore will be excluded from the study. This will be valid for each evaluation point. As stated in component 5 of the intervention, we will train FPs to support those patients excessively worried by the information given. FPs are obliged to report any side effect that occurs during the intervention. We will also evaluate the impact of the predictD-CCRT intervention on anxiety symptoms through measurements of the PRIME-MD [63,64] at each evaluation point of the follow-up.

### The intervention for the control group

Patients in the control group will receive “usual care”; that is, the kind of care that FPs usually provide when they are unaware of the risk profile for depression in the patients. This means explicitly that the patients and FPs in the control groups will not be informed about the risk profile at any point. FPs in the control group will not receive the training workshop.

## Discussion

### Ethical considerations

FPs who do not meet exclusion criteria will be asked to sign an informed consent form before health centers are allocated to the control or intervention group. Once the health centers are randomly assigned to the control or intervention groups, selected patients who do not meet exclusion criteria in the health centers of the control group will be asked for consent to follow-up and in the intervention group to receive the intervention and to follow-up. In each health center a research assistant will ask participants to sign informed consent forms. In brief, participants in the intervention group will be informed about the purpose of the trial, the expected duration of their participation, trial procedures, foreseeable risks or inconveniences, expected benefits, whom to contact for further information, their rights as participants, and that their participation is voluntary and that they may refuse to participate or withdraw from the trial at any time, without penalty or loss of benefits. Since the identity of patients will not be known at the time when randomization of health centers is to be carried out, it will not be possible to seek their consent in advance of randomization. Nevertheless, FPs will provide consent to participate in the trial; moreover, none of them will be involved as a researcher in this study, so there should not in theory be a conflict of interest. In view of the fact that so far there is no evidence that the new intervention being evaluated in this study is effective, and given that it is generally the responsibility of FPs to put the medical interests of their patients above all other considerations, this may be an appropriate choice [109].

### Strengths and limitations

To our knowledge, this is the first RCT of universal primary prevention for depression in adults and the first to test a personalized intervention implemented by FPs. We will recruit a large sample of patients and will continue the follow-up for 18 months. We will use a structured interview (CIDI) to exclude patients with major depression at baseline and to assess the cumulative incidence of major depression during follow-up. However, our study also has several limitations.

The cluster randomization by health center will minimize possible contamination bias. However, some degree of contamination could happen, since health centers of both control and intervention arms are located in the same city. This bias will be against the hypothesis of the study. The cluster randomization might produce a good balance between groups (health center); however, this does not guarantee a good balance at other levels [109] (FPs and patients). We will check at baseline the imbalance at the three levels and will adjust for it when appropriate. This and the adjustment at baseline for the likelihood of becoming depressed at 12 months will reduce our confounding bias.

As mentioned before, because we evaluate a psychosocial intervention, it is not possible for the FPs who provide it and patients that receive it to be blind [53], so FPs and patients can change their behavior because they feel observed and included in the intervention group (Hawthorne bias). FPs of the control group could also change their behavior, but it is less likely in patients from this group because they will only give their informed consent for the follow-up.

If many health centers and FPs refuse to participate, we could encounter a selection bias as the FPs who choose to participate may have a different profile (psychosocial orientation, different training and work satisfaction, etc.) to those who do not. This could limit external validity [110] and, from a pragmatic standpoint, the new intervention would be less effective when applied as a general program. Similar biases could occur if a large proportion of patients refuse to participate. It is not easy to obtain information from those physicians and patients who refuse to participate, so it will be difficult to ascertain the direction of this possible selection bias. As stated above, for the analyses with complete cases we will use the inverse probability weighting [84,85] to adjust for a possible attrition bias due to participants lost during the follow-up. We will use multiple imputations to minimize attrition bias and maintain statistical power [78,111].

The results obtained will be applicable to primary care attendees and cannot necessarily be generalized to other settings such as the general population. Nevertheless, primary care is an ideal setting for prevention and attendees are generally (because of health and social problems) at greater risk of depression than the general population.

If the predictD-CCRT intervention proves effective, it will be difficult to determine which of the 5 components are active or expendable, and how each interacts with the others. We will conduct a number of secondary analyses that could provide clues to the most relevant component, but more studies (quantitative and qualitative) specifically directed toward that goal will be needed.

## Abbreviations

FPs: Family physicians; FP: Family physician; RCT: Randomized controlled trial; CIDI: Composite international diagnostic interview; OR: Odds ratio; NNT: Number needed to treat; CSRI: Client Service Receipt Inventory; QUALYs: Quality adjusted life years; ICER: Incremental cost-effectiveness ratios; ICUR: Incremental cost-utility ratios; GLM: Generalized linear models; CEACs: Cost effectiveness acceptability curves.

## Competing interests

The authors all declare they have no competing interests.

## Authors’ contributions

JAB designed the predictD-CCRT study and the other authors collaborated in the design. JAB, LSC, JMA, CFA, CMF, CMP obtained funding for implementing the study. JAB is coordinator of the predictD-CCRT study and AF, JMA, CFA, CMF, CM, MIBR and ARB coordinate the study in each Spanish city. SCC, PMP, MK, IN, ASB, IIC, ERS, MCG, PLF, MMMG, PMG, LA, DP, MEC, FZ, DNC, JM, JMAR, ARM, and LSC collaborate implementing the study in each Spanish city. JAB, AF and JDL drafted the paper and all authors discussed and agreed the final version.

## Authors’ information

All these authors are research members of the Spanish Network of Primary Care Research (redIAPP), which is financed by the Institute of Health Carlos III (ISCIII): JAB, SCC, PMP, MK, CMP, MIBR, ARB, DNC, JMAR, ARM and JDL from “the Mental-Health, Services and Primary Care (SAMSERAP) Malaga group”; CMA, ERS and PMG from “the Castilla-Leon group”; ASB, AF, DP, JM, and LSC from “the Mental Health (SJD) Barcelona group”; JMA, MCG, and MEC from “the Bizkaya group”; and CMF, PLF and FZ from “the Aragon group”.

## Pre-publication history

The pre-publication history for this paper can be accessed here:

http://www.biomedcentral.com/1471-244X/13/171/prepub

## Supplementary Material

Additional file 1 **Annex 1.** Training workshop in the predictd-CCRT intervention: educational units. Annex 2. Primary prevention of major depression based on the level and risk profile of primary care attendees. Annex 3. A video example of the predictD-CCRT intervention. Click here for file

## References

[B1] The ESEMeD⁄MHEDEA 2000 Investigators Prevalence of mental disorders in Europe: results from the European Study of the Epidemiology of Mental Disorders (ESEMeD) project Acta Psychiatr Scand 2004 109 Suppl. 420 21 27 10.1111/j.1600-0047.2004.00327.x 15128384

[B2] Kessler RC Berglund P Denler O Jin R Koretz D Merikangas KR Rush AJ Walters EE Wang PS The epidemiology of major depressive disorder: results from the National Comorbidity Survey Replication (NCS-R) JAMA 2003 289 3095 3105 10.1001/jama.289.23.3095 12813115

[B3] King M Nazareth I Levy G Walker C Morris R Weich S Bellón JA Moreno B Svab I Rotar D Rifel J Maaroos HI Aluoja A Kalda R Neeleman J Geerlings MI Xavier M de Almeida MC Correa B Torres-González F Prevalence of common mental disorders in general practice attendees across Europe Br J Psychiatry 2008 192 362 367 10.1192/bjp.bp.107.039966 18450661

[B4] Aragones E Piñol JL Labad A Masdeu RM Pino M Cervera J Prevalence and determinants of depressive disorders in primary care practice in Spain Int J Psychiatry Med 2004 34 21 35 10.2190/C25N-W4NY-BN8W-TXN2 15242139

[B5] Luppa M Heinrich S Angermeyer MC König HH Riedel-Heller SG Cost-of-illness studies of depression: a systematic review J Affect Disord 2007 98 29 43 10.1016/j.jad.2006.07.017 16952399

[B6] Sobocki P Jönsson B Angst J Rehnberg C Cost of depression in Europe J Ment Health Policy Econ 2006 9 87 98 17007486

[B7] Mathers CD Loncar D Projections of global mortality and burden of disease from 2002 to 2030 PLoS Med 2006 3 442 10.1371/journal.pmed.0030442 PMC1664601 17132052

[B8] Ustün TB Ayuso-Mateos JL Chatterji S Mathers C Murray CJ Global burden of depressive disorders in the year 2000 Br J Psychiatry 2004 184 386 392 10.1192/bjp.184.5.386 15123501

[B9] Marquet RL Bartelds AIM Kerkhof AJFM Schellevis FG Van der Zee J The epidemiology of suicide and attempted suicide in Dutch general practice 1983–2003 BMC Fam Pract 2005 6 1 7 10.1186/1471-2296-6-1 16271136 PMC1291363

[B10] Cuijpers P Smit F Excess mortality in depression: A meta-analysis of community studies J Affect Disord 2002 72 227 236 10.1016/S0165-0327(01)00413-X 12450639

[B11] Chisholm D Sanderson K Ayuso-Mateos JL Saena S Reducing the global burden of depression: population-level analysis of intervention cost effectiveness in 14 world regions Br J Psychiatry 2004 184 393 403 10.1192/bjp.184.5.393 15123502

[B12] Von Korff M Goldberg D Improving outcomes in depression: The whole process of care needs to be enhanced BMJ 2001 323 948 949 10.1136/bmj.323.7319.948 11679372 PMC1121496

[B13] Wahlbeck K, Mäkinen M Prevention of depression and suicide. Consensus paper 2008 Luxembourg: European Communities

[B14] Pössel P Baldus C Horn AB Groen G Hautzinger M Influence of general self-efficacy on the effects of a school-based universal primary prevention program of depressive symptoms in adolescents: a randomized controlled follow-up study J Child Psychol Psychiatry 2005 46 982 994 10.1111/j.1469-7610.2004.00395.x 16109001

[B15] Spence SH Sheffield JK Donovan CL Long-term outcome of a school-based, universal approach to prevention of depression in adolescents J Consult Clin Psychol 2005 73 160 167 15709843 10.1037/0022-006X.73.1.160

[B16] Clarke GN Hornbrook M Lynch F Polen M Gale J Beardslee W O’Connor E Seeley J A randomized trial of a group cognitive intervention for preventing depression in adolescent offspring of depressed parents Arch Gen Psychiatry 2001 58 1127 1134 10.1001/archpsyc.58.12.1127 11735841

[B17] Beardslee WR Gladstone TRG Wright EJ Cooper AB A family-based approach to the prevention for depressive symptoms in Children at Risk: evidence of parental and child change Pediatrics 2003 112 e119 e131 10.1542/peds.112.2.e119 12897317

[B18] Cole MG Brief interventions to prevent depression in older subjects: a systematic review of feasibility and effectiveness Am J Geriatr Psychiatry 2008 16 6 435 443 10.1097/JGP.0b013e318162f174 18515687

[B19] van’t Veer-Tazelaar PJ van Marwijk HWJ van Oppen P Hein PJ van Hout HPJ van der Horst HE Cuijpers P Smit F Beekman ATF Stepped-care prevention of anxiety depression in late life: a randomized controlled trial Arch Gen Psychiatry 2009 66 3 297 304 10.1001/archgenpsychiatry.2008.555 19255379

[B20] Hackett ML Anderson CS House AO Halteh C Interventions for preventing depression after stroke Cochrane Database Syst Rev 2008 3 CD003689 18646094 10.1002/14651858.CD003689.pub3

[B21] Dennis CL Psychosocial and psychological interventions for prevention of postnatal depression: systematic review BMJ 2005 3031 7507 15 15994688 10.1136/bmj.331.7507.15 PMC558531

[B22] Howard LM Hoffbrand S Henshaw C Boath L Bradley E Antidepressant prevention of postnatal depression Cochrane Database Syst Rev 2005 2 CD004363 15846711 10.1002/14651858.CD004363.pub2

[B23] Brugha TS Morrell CJ Slade P Walters SJ Universal prevention of depression in women postnatally: cluster randomized trial evidence in primary care Psychol Med 2011 41 739 748 10.1017/S0033291710001467 20716383 PMC3042795

[B24] Pitceathly C Maguire P Fletcher I Parle M Tomenson B Creed F Can a brief psychological intervention prevent anxiety or depressive disorders in cancer patients? A randomized controlled trial Ann Oncol 2009 20 928 934 10.1093/annonc/mdn708 19126633

[B25] Bot M Pouwer F Ormel J Slaets JP de Jonge P Predictors of incident major depression in diabetic outpatients with subthreshold depression Diabet Med 2010 27 11 1295 1301 10.1111/j.1464-5491.2010.03119.x 20950389

[B26] Rovner BW Casten RJ Hegel MT Leiby BE Tasman WS Preventing depression in age-related macular degeneration Arch Gen Psychiatry 2007 64 886 892 10.1001/archpsyc.64.8.886 17679633

[B27] De Jonge P Hadj FB Boffa D Zdrojewski C Dorogi Y So A Ruiz J Stiefel F Prevention of major depression in complex medically ill patients: preliminary results from a randomized, controlled trial Psychosomatics 2009 50 227 233 10.1176/appi.psy.50.3.227 19567761

[B28] Mittelman MS Brodaty H Wallen AS Burns A A 3 Country Randomized Controlled Trial of a Psychosocial Intervention for Caregivers Combined with Pharmacological Treatment for Patients with Alzheimer’s Disease: Effects on Caregiver Depression Am J Geriatr Psychiatry 2008 16 11 893 904 10.1097/JGP.0b013e3181898095 18978250 PMC2753499

[B29] Muñoz RF Ying YW Bernal G Pérez-Stable EJ Sorensen JL Hargreaves WA Miranda J Miller LS Prevention of depression with primary care patients: a randomized controlled trial Am J Community Psychol 1995 23 199 222 10.1007/BF02506936 7572830

[B30] Wells K Sherbourne C Duan N Unützer J Miranda J Schoenbaum M Ettner SL Meredith LS Rubenstein L Quality improvement for depression in primary care: Do patients with subthreshold depression benefit in the long run? Am J Psychiatry 2005 162 1149 1157 10.1176/appi.ajp.162.6.1149 15930064

[B31] Allart-van Dam E Hosman CM Hoogduin CA Schaap CP Prevention of depression in subclinically depressed adults: follow-up effects on the ‘Coping with depression’ course J Affect Disord 2007 97 219 228 10.1016/j.jad.2006.06.020 16860874

[B32] Willemse GRWM Smit F Cuijpers P Tiemens BG Minimal-contact psychotherapy for sub-threshold depression in primary care Br J Psychiatry 2004 185 416 421 10.1192/bjp.185.5.416 15516551

[B33] King M Nazareth I Levy G Walker C Royston P Weich S Bellón JA Moreno-Küstner B Svab I Rotar D Rifel J Maaroos HI Aluoja A Kalda R Neeleman J Geerlings MI Xavier M Carraça I Gonçalves-Pereira M Vicente B Saldivia S Melipillan R Torres-Gonzalez F Nazareth I Development and validation of a risk prediction algorithm for episodes of major depression in European general practice attendees: the PREDICT study Arch Gen Psychiatry 2008 65 12 1368 1376 10.1001/archpsyc.65.12.1368 19047523

[B34] Bellón JA Luna JD King M Moreno-Küstner B Nazareth I Montón-Franco C GildeGómez-Barragán MJ Sánchez-Celaya M Díaz-Barreiros MÁ Vicens C Cervilla JA Svab I Maaroos HI Xavier M Geerlings MI Saldivia S Gutiérrez B Motrico E Martínez-Cañavate MT Oliván-Blázquez B Sánchez-Artiaga MS March S del Mar M-GM Vázquez-Medrano A Moreno-Peral P Torres-González F Predicting the onset of major depression in primary care: international validation of a risk prediction algorithm from Spain Psychol Med 2011 41 10 2075 2088 10.1017/S0033291711000468 21466749

[B35] Cuijpers P Prevention: an achievable goal in personalized medicine Dialogues Clin Neurosci 2009 11 4 447 454 20135902 10.31887/DCNS.2009.11.4/pcuijpers PMC3181930

[B36] Golubnitschaja O Costigliola V EPMA General Report & Recommendations in Predictive, Preventive and Personalized Medicine 2012: White Paper of the European Association for Predictive, Preventive and Personalized Medicine EPMA J 2012 3 14 http://www.epmajournal.com/content/3/14 10.1186/1878-5085-3-14 23116135 PMC3485619

[B37] Calear L Christensen H Systematic review of school-based prevention and early intervention programs for depression J Adolesc 2010 33 429 438 10.1016/j.adolescence.2009.07.004 19647310

[B38] Kavanagh J Oliver S Lorenc T Caird J Tucker H Harden A Greaves A Thomas J Oakley A School-based cognitive-behavioural interventions: A systematic review of effects and inequalities Health Sociol Rev 2009 18 61 78 10.5172/hesr.18.1.61

[B39] Neil AL Christensen H Australian school-based prevention and early intervention programs for anxiety and depression: a systematic review MJA 2007 186 305 308 17371212 10.5694/j.1326-5377.2007.tb00906.x

[B40] Stice E Shaw H Bohon C Marti N Rohde P A Meta-Analytic Review of Depression Prevention Programs for Children and Adolescents: Factors that Predict Magnitude of Intervention Effects J Consult Clin Psychol 2009 77 486 503 19485590 10.1037/a0015168 PMC2758769

[B41] Merry SN Hetrick SE Cox GR Brudevold-Iversen T Bir JJ McDowell H Psychological and educational interventions for preventing depression in children and adolescents Cochrane Database Syst Rev 2011 12 Art. No.: CD003380 10.1002/14651858.CD003380.pub3 22161377

[B42] Dennis CL Dowswell T Psychosocial and psychological interventions for preventing postpartum depression Cochrane Database Syst Rev 2013 2 CD001134 23450532 10.1002/14651858.CD001134.pub3 PMC11936315

[B43] Van der Waerden J Hoefnagels C Hosman CMH Psychosocial preventive interventions to reduce depressive symptoms in low-SES women at risk: A meta-analysis J Affect Disord 2011 128 10 23 10.1016/j.jad.2010.02.137 20346517

[B44] Cuijpers P Smit F van Straten A Psychological treatments of subthreshold depression: a meta-analytic review Acta Psychiatr Scand 2007 115 434 441 10.1111/j.1600-0447.2007.00998.x 17498154

[B45] Cuijpers P Muñoz RF Clarke GN Lewinsohn PM Psychoeducational treatment and prevention of depression: the “Coping with Depression” course thirty years later Clin Psychol Rev 2009 29 449 458 10.1016/j.cpr.2009.04.005 19450912

[B46] Cuijpers P Van Straten A Smit F Mihalopoulos C Beekman A Preventing the onset of depressive disorders: a meta-analytic review of psychological interventions Am J Psychiatry 2008 165 1272 1280 10.1176/appi.ajp.2008.07091422 18765483

[B47] Muñoz R Cuijpers P Smit F Barrera AZ Leykin Y Prevention of major depression Annu Rev Clin Psychol 2010 6 181 212 10.1146/annurev-clinpsy-033109-132040 20192789

[B48] Smit F Willemse G Koopmanschap M Onrust S Cuijpers P Beekman A Cost-effectiveness of preventing depression in primary care patients: randomised trial Br J Psychiatry 2006 188 330 336 10.1192/bjp.188.4.330 16582059

[B49] Van’t Veer-Tazelaar P Smit F van Hout H van Oppen P van der Horst J Beekman A van Marwijk H Cost-effectiveness of a stepped care intervention to prevent depression and anxiety in late life: randomised trial Br J Psychiatry 2010 196 4 319 325 10.1192/bjp.bp.109.069617 20357310

[B50] Mihalopoulos C Vos T Pirkis J Smit F Carter R Do indicated preventive interventions for depression represent good value for money? Aust NZ J Psychiatr 2011 45 1 36 44 10.3109/00048674.2010.501024 21073354

[B51] Mihalopoulos C Vos T Pirkis J The economic analysis of prevention in mental health programs Annu Rev Clin Psychol 2011 7 169 201 10.1146/annurev-clinpsy-032210-104601 21443447

[B52] Reinolds CF Prevention of depressive disorders: a brave new world Depress Anxiety 2009 26 1062 1065 10.1002/da.20644 19957277 PMC2991119

[B53] Boutron I Moher D Altman DG Schulz KF Ravaud P Extending the CONSORT Statement to Randomized Trials of Nonpharmacologic Treatment: Explanation and Elaboration Ann Intern Med 2008 148 295 309 10.7326/0003-4819-148-4-200802190-00008 18283207

[B54] Robins LN Wing JK Wittchen HU Helzer JE Babor T Burke J Farmer A Jablenski A Pickens R Regier DA Sartorius N Towle LH The Composite International Diagnostic Interview. An epidemiologic instrument for use in conjunction with different diagnostic systems and in different cultures Arch Gen Psychiatry 1988 45 1069 1077 10.1001/archpsyc.1988.01800360017003 2848472

[B55] Rubio-Stipec M Bravo M Canino G The Composite International Diagnostic Interview (CIDI): an epidemiologic instrument suitable for using in conjunction with different diagnostic systems in different cultures [Article in Spanish] Acta Psiquiatr Psicol Am Lat 1991 37 191 204 1811404

[B56] World Health Organization (WHO) Composite International Diagnostic Instrument (CIDI). Version 2.1 1997 Geneva: World Health Organization

[B57] Jenkinson C Layte R Jenkinson D Lawrence K Petersen S Paice C Stradling J A shorter form health survey: can the SF-12 replicate results from the SF-36 in longitudinal studies? J Public Health Med 1997 19 179 186 10.1093/oxfordjournals.pubmed.a024606 9243433

[B58] Gandek B Ware JE Aaronson NK Apolone G Bjorner JB Brazier JE Bullinger M Kaasa S Leplege A Prieto L Sullivan M Cross-validation of item selection and scoring for the SF-12 Health Survey in nine countries: results from the IQOLA Project. International Quality of Life Assessment J Clin Epidemiol 1998 51 1171 1178 10.1016/S0895-4356(98)00109-7 9817135

[B59] The EuroQol Group EuroQol–a new facility for the measurement of health-related quality of life Health Policy 1990 16 3 199 208 10109801 10.1016/0168-8510(90)90421-9

[B60] Badia X Schiaffino A Alonso J Herdman M Using the EuroQoI 5-D in the Catalan general population: feasibility and construct validity Qual Life Res 1998 7 4 311 322 10.1023/A:1008894502042 9610215

[B61] Badia X Roset M Montserrat S Herdman M Segura A The Spanish version of EuroQol: a description and its applications. European Quality of Life scale. [Article in Spanish] Med Clin (Barc) 1999 112 Suppl 1 79 85 10618804

[B62] Bellón JA Moreno B Torres-González F Montón-Franco C GildeGómez-Barragán MJ Sánchez-Celaya M Díaz-Barreiros MA Vicens C Luna JD Cervilla JA Gutierrez B Martínez-Cañavate MT Olivan-Blázquez B Vázquez-Medrano A Sánchez-Artiaga MS March S Motrico E Ruiz-García VM Brangier-Wainberg PR Muñoz-García MD Nazareth I King M predict D group Predicting the onset and persistence of episodes of depression in primary health care. The predictD-Spain study: methodology BMC Pub Health 2008 8 256 10.1186/1471-2458-8-256 18657275 PMC2527330

[B63] Spitzer RI Kroenke K Williams JBW Validation and utility of a self-report version of PRIME-MD: The PHQ Primary Care Study JAMA 1999 282 1737 1744 10.1001/jama.282.18.1737 10568646

[B64] Baca E Saiz J Agüera L Caballero L Fernández-Liria A Ramos J Gil A Madrigal M Porras A Validation of the Spanish version of PRIME-MD: a procedure for diagnosing mental disorders in primary care. [Article in Spanish] Actas Españolas de Psiquiátricas 1999 27 375 383 10611561

[B65] Karasek R Theorell T Healthy work stress, productivity and the reconstruction of working life 1990 New York: Basic Books

[B66] Arroll B Khin N Kerse N Screening for depression in primary care with two verbally asked questions: cross sectional study BMJ 2003 327 1144 1146 10.1136/bmj.327.7424.1144 14615341 PMC261815

[B67] Fink LA Bernstein D Handelsman L Foote J Lovejoy M Initial reliability and validity of the childhood trauma interview: a new multidimensional measure of childhood interpersonal trauma Am J Psychiatr 1995 152 1329 1335 7653689 10.1176/ajp.152.9.1329

[B68] Qureshi N Bethea J Modell B Brennan P Papageorgiou A Raeburn S Hapgood R Modell M Collecting genetic information in primary care: evaluating a new family history tool Fam Pract 2005 22 663 669 10.1093/fampra/cmi073 16055464

[B69] Sproston K Primatesta P Health Survey for England. Volume 1: The health of children and young people 2003 London: The Stationery Office

[B70] Janssen I Hanssen BM Bijil RV De Graaf R Vollebergh W McKenzie K Van Os J Discrimination and ideation Br J Psychiatry 2003 182 71 76 10.1192/bjp.182.1.71 12509322

[B71] King M Weich S Torres F Svab I Maaroos H Neeleman J Xavier M Morris R Walker C Bellon JA Moreno B Rotar D Rifel J Aluoja A Kalda R Geerlings MI Carraca I Caldas de Almeida M Vicente B Saldivia S Rioseco P Nazareth I Prediction of depression in European general practice attendees: the PREDICT study BMC Public Health 2006 6 1 6 10.1186/1471-2458-6-6 16409633 PMC1368984

[B72] Mira JJ Llinás G Gil V Orozco D Palazón I Villater J Validation of an instrument for identifying styles of the professional practice of the primary care doctor [Article in Spanish] Aten Primaria 1998 21 14 22 9557352

[B73] Francis LJ Brown LB Philipchalk R The development of an abbreviated form of the Revised Eysenck Personality Questionnaire (EPQR-A): Its use among students in England, Canada, the U.S.A and Australia Pers Indiv Differ 1992 1 443 449

[B74] Forrest S Lewis CA Shevlin M Examining the factor structure and differential functioning of the Eysenck Personality Questionnaire Revised-Abbreviated Pers Indiv Differ 2000 29 579 588 10.1016/S0191-8869(99)00220-2

[B75] Sandin B Valiente RM Chorot P Olmedo M Santed MA Spanish versión of the Eysenck Personality Questionnaire-Revised (EPQR-A) (I): Exploratory factor analysis [Article in Spanish] Revista de Psicopatología y Psicología Clínica 2002 7 3 195 205

[B76] Groenwold RHH Donders ART Roes KCB Harrell FE Jr Moons KGM Dealing With Missing Outcome Data in Randomized Trials and Observational Studies Am J Epidemiol 2012 175 210 217 10.1093/aje/kwr302 22262640

[B77] Corporation STATA Stata Statistical Software, Release 12 2011 Texas: College Statation

[B78] White IR Royston P Wood AM Multiple imputation using chained equations: Issues and guidance for practice Statist Med 2011 30 377 399 10.1002/sim.4067 21225900

[B79] Royston P Multiple imputation of missing values: update of ice Stata J 2005 5 527 536

[B80] Graham JW Olchowski AE Gilreath TD How many imputations are really needed? Some practical clarifications of multiple imputation theory Prev Sci 2007 8 206 213 10.1007/s11121-007-0070-9 17549635

[B81] Rubin D Multiple imputation for nonresponse in surveys 1987 New York: Wiley

[B82] White IR Carlin JB Spratt M Royston P Kenward MG Wood A Carpenter JR Multiple imputation for missing data in epidemiological and clinical research: potential and pitfalls BMJ 2009 338 b2393 10.1136/bmj.b2393 19564179 PMC2714692

[B83] Snijders TAB Bosker RJ Multilevel Analysis. An Introduction to Basic and Advanced Multilevel Modelling 1999 London: Sage Publications

[B84] Hernán MA Hernández-Díaz S Robins JM A structural approach to selection bias Epidemiology 2004 15 615 625 10.1097/01.ede.0000135174.63482.43 15308962

[B85] Bellón JA Luna JD Moreno B Montón-Franco C Gildegómez-Barragán MJ Sánchez-Celaya M Díaz-Barreiros MA Vicens C Motrico E Martínez-Cañavate MT Olivan-Blázquez B Vázquez-Medrano A Sánchez-Artiaga MS March S Muñoz-García MD Moreno-Peral P Nazareth I King M Torres-González F Psychosocial and sociodemographic predictors of attrition in a longitudinal study of major depression in primary care : the predictD-Spain study J Epidemiol Community Health 2010 64 874 884 10.1136/jech.2008.085290 19759057

[B86] Rabe-Hesketh S Skrondal A Multilevel and Longitudinal Modeling Using Stata. Volumen II: Categorical Responses, Counts, and Survival 2012 3 College Station, Texas: STATA Press

[B87] National Institute for Health and Clinical Excellence Guide to the Methods of Technology Appraisal 2008 London: NICE Update: http://www.nice.org.uk/media/955/4F/Clarification_to_section_5.6_of_the_Guide_to_Methods_of_Technology_Appraisals.pdf 27905712

[B88] Steward WF Ricci JA Chee E Hahn SR Morganstein D Cost of lost productive work time among US workers with depression JAMA 2003 289 3135 3144 10.1001/jama.289.23.3135 12813119

[B89] Salvador-Carulla L Bendeck M Fernández A Alberti C Sabes-Figuera Molina C Knapp M Cost of depression in Catalonia (Spain) J Affect Disord 2011 132 1–2 130 138 21402411 10.1016/j.jad.2011.02.019

[B90] Knapp M PSSRU and Centre for Economics of Mental Health IoP, University of Kent Economic Evaluation of Mental Health Care 1995 UK: Ashgate Publishing Group

[B91] Herdman M Badia X Berra S EuroQol-5D: a simple alternative for measuring health-related quality of life in primary care [Article in Spanish]. Aten Primaria 2001 28 425 429 10.1016/S0212-6567(01)70406-4 11602124 PMC7684037

[B92] Herdman M Badia X Berra S EuroQol-5D: a simple alternative for measuring health-related quality of life in primary care [Article in Spanish] Aten Primaria 2001 28 425 429 10.1016/S0212-6567(01)70406-4 11602124 PMC7684037

[B93] Glick HA Doshi JA Sonnad SS Polsky D Economic evaluation in clinical trials 2007 Oxford: Oxford University Press

[B94] Efron B Missing data, imputation, and the bootstrap J Am Stat Assoc 1994 89 463 475 10.1080/01621459.1994.10476768

[B95] Fenwick E Byford S A guide to cost-effectiveness acceptability curves Br J Psychiatry 2005 187 106 108 10.1192/bjp.187.2.106 16055820

[B96] Medical Research Council A framework for development and evaluation of RCTs for complex interventions to improve health 2000 London: MRC

[B97] Moreno-Peral P Bellón JA Motrico E Moreno-Küstner B Oliván-Blázquez B Fernández A Fernández-Alonso C Rüntel A Rodríguez-Bayón A Ballesta-Rodríguez MI Payo-Gordón J Amezcua M Primary prevention in depression: How to inform primary care attendees about their risk level and risk profile of major depression? Eur Psychiatry 2010 25 Suppl.1 922

[B98] Gigerenzer G Edwards A Simple tools for understanding risks: from innumeracy to insight BMJ 2003 327 741 744 10.1136/bmj.327.7417.741 14512488 PMC200816

[B99] Halvorsen PA Selmer R Kristiansen IS Different ways to describe the benefits of risk-reducing treatments Ann Intern Med 2007 146 848 856 10.7326/0003-4819-146-12-200706190-00006 17577004

[B100] Jorm AF Christensen H Griffiths KM The public's ability to recognize mental disorders and their beliefs about treatment: changes in Australia over 8 years Aust NZ J Psychiatr 2006 40 36 41 10.1080/j.1440-1614.2006.01738.x 16403035

[B101] Stuart MR III Lieberman JA The fifteen minute hour: Applied psychotherapy for the primary care physician 1993 2 Westport: Praeger Publishers

[B102] McDaniel S Campbell TL Hepworth J Lorenz A Satcher D Family-Oriented Primary Care. A manual for medical provider 2004 2 New York: Springer-Verlag

[B103] Doherty WJ Baird MA Development levels in family-centered medical care Fam Med 1986 18 153 156 3582830

[B104] Cawston P Social prescribing in very deprived areas: “I didn’t become a GP to spend my life prescribing pills” Br J Gen Pract 2011 61 350 10.3399/bjgp11X572517 21619768 PMC3080219

[B105] Dowrick C May C Richardson M Bundred P The biopsychosocial model of general practice: rhetoric or reality? Br J Gen Pract 1996 46 105 107 8855018 PMC1239540

[B106] Fernández-Alonso C Buitrago F Ciurana R Chocrón L García-Campayo J Montón C Tizón JL Prevention of mental disorders [Article in Spanish] Aten Primaria 2012 44 Suppl 1 52 56 23399507 10.1016/S0212-6567(12)70014-8 PMC8171406

[B107] Library of Clinical Practice Guidelines of the National Health System, guiasalud.es http://portal.guiasalud.es/web/guest/informacion-pacientes

[B108] Schomerus G Angermeyer MC Matschinger H Riedel-Heller SG Public attitudes towards prevention of depression J Affect Disord 2008 106 257 263 10.1016/j.jad.2007.06.013 17673299

[B109] Hayes RJ Moulton LH Cluster Randomized Trials 2009 NewYork: Chapman & Hall/CRC

[B110] Eldridge S Ashby D Bennett C Wakelin M Feder G Internal and external validity of cluster randomized trials: systematic review of recent trials BMJ 2008 336 876 880 10.1136/bmj.39517.495764.25 18364360 PMC2323095

[B111] Bell ML Kenward MG Fairclough DL Horton NJ Differential dropout and bias in randomized controlled trials: when it matters and when it may not BMJ 2013 346 e8668 10.1136/bmj.e8668 23338004 PMC4688419

