# Supplementary material for: Preventing the onset of major depression based on the level and profile of risk of primary care attendees: protocol of a cluster randomised trial (the predictD-CCRT study)
Source: BMC Psychiatry. 2013 Jun 19;13:171. doi: 10.1186/1471-244X-13-171 (PMC3698147; doi:10.1186/1471-244X-13-171)
Supplement: Additional file 1 — Annex 1. Training workshop in the predictd-CCRT intervention: educational units. Annex 2. Primary prevention of major depression based on the level and risk profile of primary care attendees. Annex 3. A video example of the predictD-CCRT intervention. [file 1471-244X-13-171-S1.doc]

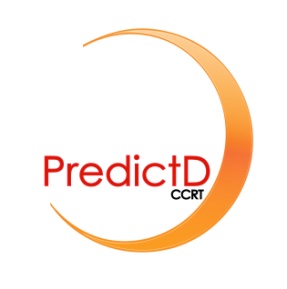
 **ANNEX 1**

**TRAINING WORKSHOP IN THE PREDICTD-CCRT INTERVENTION: EDUCATIONAL UNITS**

- - **Unit 1:** Risk factors for depression
  - **Unit 2:** The predictD study and the predictD-CCRT project
  - **Unit 3:** Evidence for promotion and primary prevention of depression
  - **Unit 4:** The intervention predictD-CCRT
  - **Unit 5:** Preventing the onset of major depression in primary care patients through the predictD-CCRT intervention (Clinical cases I)
  - **Unit 6:** Preventing the onset of major depression in primary care patients through the predictD-CCRT intervention (Clinical cases II)
  - **Unit 7:** Preventing the onset of major depression in primary care patients through the predictD-CCRT intervention (Clinical cases III)
  - **Unit 8:** Preventing the onset of major depression in primary care patients through the predictD-CCRT intervention (Clinical cases IV)
  - **Unit 9:** Update: clinical presentation, diagnosis and treatment of depression and anxiety disorders in primary care.
  - **Unit 10:** Preventing recurrences and relapses of episodes of major depression


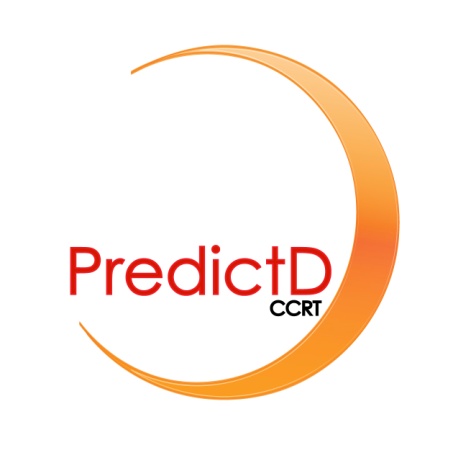


**ANNEX 2**

**PRIMARY PREVENTION OF MAJOR DEPRESSION BASED ON THE LEVEL AND RISK PROFILE OF PRIMARY CARE ATTENDEES**

Your family physician has told you about your risk of depression in the next 12 months. It is important that you understand that you do NOT have depression now. However, if your doctor has told you that your risk for having depression is either low, moderate or high, there are several things you can do to help prevent depression. So, please read this brochure carefully and feel free to talk to your doctor about it.

**RECOMMENDATIONS TO IMPROVE MENTAL HEALTH**

Two aspects have to be taken into account to look after mental health:

- **Look after yourself**
- **Look after your relationships with others**

**LOOKING AFTER YOURSELF**

Disregarding our **obligation to look after ourselves** is a source of unease, stress, frustration and becoming dispirited.

- **Physical exercise**
- Doing regular physical exercise has been shown to increase life expectancy and help maintain physical and mental health.
- It is a good idea to incorporate this exercise into our daily lives, by walking, going up the stairs, cycling …
- Doing about 30 minutes of moderate physical exercise on most days of the week is enough to obtain the associated health benefits.
- Older persons or those with some disability can improve such aspects as balance, strength, and their disability if they do exercises adapted to their personal situation.
- **Diet**
- This should be varied and adequate in energy intake (calories) to maintain an adequate weight.
- Reduce the consumption of animal fats and sweet things, as well as drinks containing caffeine and the excessive consumption of alcoholic drinks.
- It is recommended to have your meals at the same time each day and take your time to enjoy them.
- **Sleep hygiene**
- Adequate rest benefits our mental health
- Go to bed and get up at the same times each day
- An adult has enough sleep with 7-8 hours every night, though this can vary from person to person.
- It is a good idea to do some light relaxing activity during the hour before going to bed (reading, listening to music …)
- If you have problems sleeping, it is not a good idea to listen to the radio or watch TV in bed before going to sleep.
- Avoid drinking any drinks containing caffeine or alcohol.
- Ensure the atmosphere in the bedroom is adequate. The room should be dark, quiet, previously aired and at a suitable temperature.
- If you have a midday nap, it should be no longer than 30 minutes.
- **Enjoy your free time**, doing **leisure activities that you like** to make you feel better.

**LOOKING AFTER YOUR RELATIONSHIPS WITH OTHERS**

- **Share your problems** with other persons who have experienced the same sort of situation. This will help you find a solution to your conflict and make you feel less alone.
- **Dedicate time to your family and friends**, building up these relationships and finding the time needed for it.
- Try to **make new friends and keep your existing ones**. Friendship means an exchange: support others and in turn, you will receive their support.
- **Put your worries at work in perspective**. Try to “disconnect” at the end of the work day.

If you want more information we recommend the following brochures for patients

**Depression: information for the patient, the family and other interested persons.**

<http://www.guiasalud.es/GPC/GPC_424_Dep_Adult_paciente.pdf>

**Learning to recognise and cope with anxiety: generalised anxiety and distress. Information for patients.**

<http://www.guiasalud.es/GPC/GPC_430_Ansiedad_Lain_Entr_paciente.pdf>

**Learning to recognise and cope with insomnia. Information for patients.**

<http://www.guiasalud.es/GPC/GPC_465_Insomnio_Lain_Entr_paciente.pdf>

**ANNEX 3**

The link below shows a short 5-minute video with a simulated case of the predictD-CCRT intervention.

<http://www.youtube.com/watch?v=kbFhVVDNFGE>

**PredictD information (level and risk profile of depression): Risk score 21%,** A 53-year-old woman, living in Malaga (Spain)**;** personal history of depression, suffered physical childhood abuse, does not take medication for anxiety, depression or stress, SF-12 mental score 40 and SF-12 physical scale score 62, one serious problem in very close persons**,** satisfied with unpaid work and satisfied with living together at home.

**Information accessible to the family physician from the clinical chart:** Katia is a foreigner from Romania who has been in Spain for 3 years. One week ago she went to see her family physician due to breast pain. In her left breast the family doctor found three minor nodules of 0.5 cm, non adherent to surrounding tissues. No nipple secretion, no axillary, lateral-cervical or supraclavicular adenopathies. The patient was then overtly worried (afraid of breast cancer) and her family physician ordered a mammography.
